# PROTOCOL: Health and social care interventions in the 80 years old and over population: An evidence and gap map

**DOI:** 10.1002/cl2.1326

**Published:** 2023-05-09

**Authors:** Rebecca Abbott, Jo Thompson Coon, Alison Bethel, Morwenna Rogers, Rebecca Whear, Noreen Orr, Ruth Garside, Victoria Goodwin, Aseel Mahmoud, Ilianna Lourida, Debbie Cheeseman

**Affiliations:** ^1^ NIHR ARC South West Peninsula (PenARC) University of Exeter Medical School Exeter UK; ^2^ Knowledge Spa, Royal Cornwall Hospital University of Exeter Medical School Truro UK; ^3^ Royal Devon and Exeter NHS Trust Royal Devon & Exeter Hospital Exeter UK

## Abstract

This is the protocol for a Campbell systematic review. The objectives are as follows: identify available systematic reviews and randomised controlled trials on interventions targeting health or social needs of the people aged over 80; identify qualitative studies relating to the experiences of people aged over 80 of interventions that target their health or social needs; identify areas where systematic reviews are needed; identify gaps in evidence where further primary research is needed; assess equity considerations (using the PROGRESS plus criteria) in available systematic reviews, randomised trials and qualitative studies of identified interventions; assess gaps and evidence related to health equity.

## 1 BACKGROUND

### 1.1 The problem, condition or issue

World‐wide, the population is aging. The proportion of older adults aged 65 years or older increased in most countries over the past decade and this rise is expected to continue (Jaul & Barron, 2017). Moreover, the World Health Organisation (2022) predict that the number of older adults aged 80 years and above globally has been forecast to triple between 2020 and 2050 and expected to reach 426 million. In the UK alone, those aged 80 years or more is one of the fastest growing age groups and this is set to double to 6.4 million by 2045 and treble by 2070 (ONS, 2018). The oldest among older adults have been termed the ‘oldest old’ and this is the population we are interested in. Several definitions have been proposed for this age group and while there is no consensus, over 80 years and over 85 years appear to be the two most common age cut‐offs used (Escourrou et al., 2020).

As the population ages, so does the prevalence of age‐related diseases such as arthritis, osteoporosis, diabetes, hypertension, cancer and dementia. For example for cancer incidence rates, in the UK in 2016–2018, across each year more than a third of new cases were in people aged 75 and over (Cancer Research UK, 2021). Furthermore, the likelihood of developing multiple long‐term conditions (having two or more long‐term physical or mental health conditions) increases with age, and this is reported to be globally increasing across lower, middle and high income countries (The King's Fund, 2021). The prevalence of disability also increases with age: a longitudinal study of those over 90 years in the US found difficulties in activities of daily living were present in over 75% of adults aged 90 or more, increasing to 97% in those over 100 years, and that needing help with activities of daily living was present in 44% and 92% respectively (Berlau et al., 2009). An ageing population is likely to place greater demands on health services, as evidenced by reports of a 50% increase in people over the age of 75 years being treated in English hospital in 2014–2015 compared with 2006–2007 (Lin et al., 2016). In Australia, women aged 85 years or more representing the largest proportion of emergency surgical admissions (AIHW, 2014).

There are also issues relating to age‐related health conditions for those living independently. Recent WHO estimates suggest that the proportion of older adults with significant and moderate loss of functional ability is two times greater among adults aged 80 years and over than those aged 60–70 years (WHO, 2020a). Furthermore, in the UK, over half of all people aged 75 and over live alone and it is predicted that between 2008 and 2031 the increase in those aged 75 years and over living alone will be 38% (ONS, 2010). As such, many older people will have a reduction in functional ability and require support, which if not provided by someone living with them (such as an unpaid carer, i.e., spouse or child), will need to be provided by formal services, unless they can optimise their function such that support is not required. All older adults, irrespective of the level of intrinsic capacity, should have opportunities to optimise functional ability to enjoy what they value most.

The evidence underpinning the treatments and interventions for the majority of health and social care issues is likely to have been derived from populations younger than 80 years of age. This is most probably due to older adults, particularly the oldest old, being excluded from trials due to their comorbidities (Benetos et al., 2019), or the nature of a changing demographic itself, such that the oldest old were a much smaller group when the primary research was being undertaken. There have been concerns raised that both the effectiveness and the suitability of many established treatments may not be either suitable, or the most effective approach, for the oldest old. For example, it has been suggested that whilst meta‐analyses of effectiveness trials widely used to inform evidence‐based practice can provide useful insights to average effects of interventions, they often are not able to offer robust conclusions for sub‐groups, such as those in the oldest age categories (Clegg et al., 2022).

In terms of social care, Morgan et al. (2020) reported that there is a growing recognition of the number of family caregivers who are older adults themselves living with complex health conditions, and that the research on this at‐risk group has predominantly examined the experience of caregivers aged between 60 and 75 with little known about the increasing number of caregivers who are over‐75. Morgan et al. (2020) also highlighted the increased risk for the oldest old in terms of health issues and declining social networks, and the impact this could have on both care‐giving and the care‐giver.

### 1.2 The intervention

Healthy ageing is relevant to everyone, not just those who are currently free of disease. The WHO defines healthy ageing as the process of developing and maintaining the *functional ability* that enables well‐being in older age (Rudnicka et al., 2020). The WHO suggest that functional ability is ‘determined by the intrinsic capacity of the individual (i.e., the combination of all the individual's physical and mental capacities), the environments he or she inhabits (understood in the broadest sense and including physical, social and policy environments), and the interaction between these’ (WHO, 2020a, p. 1).

As such, we are interested in interventions that impact functional ability: either directly on the intrinsic capacity of an individual, or on functional ability more broadly through health and social care interventions. We will take this health systems perspective in our consideration of interventions, broadening health care to include social care interventions that work together to impact functional ability. We will also consider determinants of health inequity.

### 1.3 Why it is important to develop the evidence and gap map (EGM)

The population aged 80 years and over is growing alongside increasing research on this population (Gonzalez‐Alcaide et al., 2021). The number of publications per year focussed on the topic of the ‘oldest old’ has steadily increased for over 3 decades (Lund & Wang, 2020). In addition to focusing on the pathologies causing the greatest mortality and morbidity in this population, such as dementia, health research in those aged 80 years and over appears to be tackling a myriad of interlinked factors, such as geriatric syndromes (such as frailty, falls, incontinence, skin breakdown), social aspects, and factors related to preserving quality of life and promoting healthy aging. However, it is unclear what research, in terms of scale and focus, is being undertaken in this population.

Due to the rapid increase of research in the oldest age groups over the past three decades, an EGM of available evidence will therefore help identify where there is sufficient knowledge to undertake a systematic review, but more likely in this area, where more evidence is needed.

## 2 OBJECTIVES

The objectives of this EGM are therefore to:

- Identify available systematic reviews and randomised controlled trials on interventions targeting health or social needs of the people aged over 80.
- Identify qualitative studies relating to the experiences of people aged over 80 of interventions that target their health or social needs.
- Identify areas where systematic reviews are needed.
- Identify gaps in evidence where further primary research is needed.
- Assess equity considerations (using the PROGRESS plus criteria) in available systematic reviews, randomised trials and qualitative studies of identified interventions.
- Assess gaps and evidence related to health equity.

*Specific research questions*

- What is the scale and focus of interventions in health and social care designed for or evaluated on those aged 80 years and over?
- What is the nature and breadth of qualitative evidence relating to how those aged 80 years and over perceive interventions aimed at their health and social wellbeing?
- Is there evidence or gaps in the research in this population related to health equity?

## 3 METHODS

### 3.1 EGM: Definition and purpose

EGMs are used to highlight what research on a topic is available alongside highlighting gaps in research to inform strategic health and social policy, program and research priorities (Welch et al., 2021). EGMs can identify areas for which there are no or few primary studies, or many studies, but no systematic reviews and can also highlight areas in which there are many reviews to indicate where a review of reviews may be appropriate (White et al., 2020). The purpose of EGMs is to allow users to identify and access the research evidence (or evidence gaps) most relevant to their population and intervention focus. We will undertake a five‐stage process:

- Agree/define a framework.
- Identify the available evidence.
- Appraise the quality of the evidence.
- Extract, code and summarise the data that relate to the objectives.
- Visualisation and presentation of the findings in a user‐friendly manner.

We will use the Campbell Collaboration mapping tool developed by the EPPI‐Centre (https://www.theepicentre.co.uk/) to display identified studies using the framework described below.

### 3.2 Framework development and scope

We could find no existing widely accepted framework that was relevant to the breadth of health and social care interventions that could have been researched for this particular age group. We decided that we would use the definition of healthy ageing and its concepts relating to intrinsic capacity, the environment and the interaction between the two to inform the basis of our framework for this EGM (WHO, 2020b). The WHO definitions for functional ability, intrinsic capacity and the environment are provided below:

- Functional ability is defined as ‘all the health‐related attributes that enable people to be and to do what they have reason to value’ (p. 2). Five sub‐domains are proposed: meeting basic needs, learning and making decisions; mobility; building and maintaining relationships; and contributing to families, communities or society.
- Intrinsic capacity at any point in time is ‘determined by many factors, including underlying physiological and psychological changes, health‐related behaviours and the presence or absence of disease’ (p. 2). Five sub‐domains are proposed: neuromusculoskeletal, sensory, metabolic, cognitive and psychological.
- Environments ‘that people inhabit and their interaction with them are also major determinants of what older people with a given level of intrinsic capacity can do. These environments provide a range of resources or barriers that will ultimately decide whether older people can engage or participate in activities that matter to them’ (p. 2). Five sub‐domains are proposed: products and technology, natural and built environment; support and relationships; attitudes; and services, systems and policies.

We will further define the scope of the framework in consultation with the wider research team with input from public and patient engagement (PPIE), practitioners, information specialists, and researchers.

The EGM framework will inform the inclusion and exclusion criteria of the EGM.

### 3.3 Stakeholder engagement

We will work with a range of stakeholders to define the scope of the EGM and help develop the framework with respect to identifying relevant interventions and outcomes. Discussion with older adults who are part of the PenARC patient engagement group has already informed the development of the protocol, and we will return for further discussions with this group when we have our initial studies to obtain their thoughts on the framework for the map. A formal Advisory Group will also work with us on the EGM, meeting at two key points in the project: identifying the EGM dimensions, and ensuring the final map makes sense and can be easily understood. The advisory group consists of: two members of the public (recruited through the Peninsula Public Engagement Group (PenPEG), a community dietitian (to be confirmed), a geriatrician, and a speech therapist.

### 3.4 Conceptual framework

We will use the definition of healthy ageing and its concepts relating to intrinsic capacity, the environment and the interaction between the two to inform the basis of our framework for this EGM (WHO, 2020a). Alongside this we will consider the multidimensional model of healthy aging, proposed by Rivadeneira et al. (2021), which is also based on the central role of functional ability in healthy aging. These authors suggest that intrinsic capacity covers the concepts of (1) physiological and metabolic health, (2) geriatric syndromes, (3) risk factors, (4) physical capacity, (5) cognitive capacity, and (6) psychological well‐being. We will adapt and revise the framework on the basis of expert review and stakeholder consultation in the early piloting stages.

### 3.5 Dimensions

The following interventions and outcomes/experiences *will be refined* after consultation with stakeholders.

*Interventions (rows in the map)*.

Interventions will be broadly categorised as those that fall under ‘building and maintaining intrinsic capacity’ and those that fall under ‘enabling environments and technologies’. Within this, interventions will be grouped:

- Building and maintaining intrinsic capacity
  ∘ Pharmaceutical (e.g., medication review, medication initiation, deprescribing, dose, drug effectiveness)
  ∘ Physical (e.g., rehabilitation, occupational health, physical activity & exercise)
  ∘ Surgical (e.g., joint surgery, cardiovascular)
  ∘ Oral/Nutritional (e.g., oral health, supplements)
  ∘ Cognitive Health (e.g., cognitive training)
  ∘ Psychological (e.g., mindfulness, cognitive behaviour)
- Enabling environments and technology
  ∘ Meeting basic needs (e.g., Palliative Care, Person‐centred Care)
  ∘ Health service models (e.g., hospital at home, CGA)
  ∘ Technology (e.g., mobile healthcare delivery, telehealth, wearables, remote monitoring, remote screening programmes)
  ∘ Physical environment (e.g., home safety modifications, mobility assistance)
  ∘ Building and maintaining relationships (e.g., carer support, volunteer scheme, intergenerational activities, animal interventions)
*Outcomes/experiences (columns in the map*)
  ∘ Experiences of interventions (e.g., experience of procedure, activity, medicine, technology) from either participants, family members, health care professionals and relevant stakeholders^1^
  ∘ Medication related (e.g., medicine optimisation/deprescribing rates/adherence)
  ∘ Physiological health (e.g., blood pressure, fitness, bone density, BMI, strength)
  ∘ Functional/physical health (e.g., activities of daily living, mobility)
  ∘ Physiological events (e.g., myocardial infarction, stroke, falls)
  ∘ Change in long term condition (e.g., frailty indices)
  ∘ Cognitive health (e.g., cognition, memory)
  ∘ Mental health and wellbeing (e.g., life satisfaction, wellbeing, loneliness, anxiety, depression)
  ∘ Resource use (e.g., hospital admission, care home admissions, primary care visits, cost)
  ∘ Social health (e.g., connectedness, participation)
  ∘ Process‐related (e.g., acceptability)
  ∘ Adverse events

### 3.6 Types of study design

We are interested in effectiveness and experience of interventions to inform decision making and to inform future research. We will therefore include systematic reviews, randomised controlled trials and qualitative studies related to relevant (health and social care) interventions.

#### 3.6.1 Systematic review

Systematic reviews may seek to evaluate RCTs, non‐randomised controlled trials, controlled and uncontrolled before‐and‐after trials, interrupted time series designs or be a qualitative synthesis relating to an intervention. All systematic reviews, irrespective of AMSTAR quality (Shea et al., 2017), will be included. To be included as a systematic review, the review needs to report (i) a research question, (ii) search sources and a reproducible search strategy, (iii) inclusion and exclusion criteria, and (iv) selection methods (adapted from Krnic Martinic et al., 2019). We will *exclude* scoping reviews, narrative reviews or any type of evidence synthesis described as a review (systematic or not) that does not fulfil the four criteria described above.

#### 3.6.2 Randomised controlled trials

We will include randomised trials on interventions targeting health or social needs of the people aged over 80 (see inclusion criteria above), and qualitative studies relating to such interventions. We will exclude quantitative studies and reviews that focus on predictive factors, prognostic and diagnostic studies. Quantitative studies that are not randomised controlled trials will be excluded.

#### 3.6.3 Qualitative studies

We will include qualitative studies that evaluate participant experience of interventions, but also clinician, family member or other relevant stakeholders perception/experience of interventions. The qualitative study does not have to be part of an RCT. We will exclude qualitative studies that are not focussed on the experience/perception of interventions.

We will also include on‐going systematic reviews and randomised trials. We will also include studies published in grey literature such as reports, dissertations, and conference abstracts, if they meet our study design criteria.

### 3.7 Types of intervention/problem

We are interested in any health or social care intervention targeted at individuals aged 80 years or more. As noted above in the dimension section above, we expect the interventions to be heterogenous, but all will target one or more of the three dimensions relating to healthy aging: intrinsic capacity, functional ability or the environment.

### 3.8 Types of population (as applicable)

We will include studies if they involve participants aged 80 years old or older. We will include a study with a wider age range if the mean age is over 80 years, or for qualitative studies, if the majority of the participants are 80 years old or more. For qualitative studies of carers of those and health or social care professionals delivering interventions to those aged 80 years or more.

### 3.9 Types of outcome measures (as applicable)

All outcomes relating to any eligible health and social care intervention are of interest. Possible outcomes have been suggested in the dimension section above. Outcomes will be extracted and presented as described in the included articles.

### 3.10 Other eligibility criteria

Due to the increased aging demographic over recent decades, we are restricting eligibility to publications since 1990. All articles before 1990 will be excluded.

The review will not be restricted by geographical area.

### 3.11 Types of settings

We will include interventions in any setting. Settings could be the individual's place of residence (such as residential homes, apartments, long‐term care facilities, hospices, nursing homes), but could also be in acute/sub‐acute hospital and convalescent care settings. We will code the settings so that the evidence can be filtered according to setting.

The full inclusion and exclusion criteria in the form of a ‘Participant, Intervention, Comparator, Outcome’ (PICO) structure are shown in Table 1.

**Table 1:** ‘80 Plus’ evidence and gap map inclusion and exclusion criteria.

Criteria	Specification
Population	Include if: ∘ Participants aged 80 years old or older ✓ we will include a study with a wider age range if the mean age is over 80 years ✓ *If review/study refers to oldest old, very old, frail and old but no age is cited in abstract, include at Ti & Ab screening stage* FOR QUALITATIVE STUDIES ∘ Carer of participants aged >80 years ∘ Health or social care professionals delivering interventions to adults > 80 years Exclude if: ∘ Studies of older adults <80 years ∘ Studies that report outcomes/experiences for a sub‐group aged 80 years or more
Intervention	Include if: ∘ Any health or social care intervention including, but not limited to: ✓ Pharmacological ✓ Physical (e.g., rehab, exercise) ✓ Surgical (e.g., joint, cardiovascular) ✓ Oral/Nutritional (e.g., oral health, supplements, food delivery) ✓ Technological (e.g., mobile healthcare, telehealth, wearables) ✓ Cognitive (e.g., cognitive training) ✓ Social/Mental Health (e.g., peer support, social connection, volunteer) ✓ Environmental (e.g., transport, home safety) ∘ Intervention must target individuals >80 years of age. Exclude if: ∘ Not targeted at individuals over 80 years ∘ Intervention is not related to health or social care.
Comparator	For RCTS only: ∘ Studies must include a comparator group that some participants are randomly allocated to. ∘ Comparator may include any type of control group or treatment comparator.
Outcomes or Experiences	Include:
	Experiences ∘ Experiences or perceptions of interventions (e.g., experience of procedure, activity, medicine, technology) from either participants, family members, health or social care professionals and/or relevant stakeholders. Outcomes ∘ Outcome measures related to health or social care, such as: ✓ Medication related (e.g., medicine optimisation/deprescribing rates/adherence) ✓ Physiological health (e.g., blood pressure, fitness, bone density, BMI, strength) ✓ Functional/physical health (e.g., activities of daily living, mobility) ✓ Physiological events (e.g., myocardial infarction, stroke, falls) ✓ Change in long‐term condition (e.g., frailty indices) ✓ Cognitive health (e.g., cognition, memory) ✓ Mental health and wellbeing (e.g., life satisfaction, wellbeing, loneliness, anxiety, depression) ✓ Resource use (e.g., hospital admission, care home admissions, primary care visits, cost) ✓ Social health (e.g., connectedness, participation) ✓ Process‐related (e.g., acceptability) ✓ Adverse events Exclude if: ∘ Qualitative data not related to intervention, for example, experience of being old
Study design	Include if: ∘ Systematic Review of any study design that meets 4 criteria: clear RQ, search sources & reproducible search strategy, incl & excl criteria, and selection methods ∘ Randomised controlled trial ∘ Qualitative study Exclude: ∘ Scoping review, or ‘review’ without methods ∘ Protocols for SR/RCT ∘ Conference proceedings ∘ Books/Chapters ∘ Editorials/Opinion Pieces/Letters ∘ A quantitative primary study but NOT an RCT (i.e., non‐randomised controlled trial, retrospective cohort study, cross sectional analysis).
Other	Include if: ∘ Published >1990 ∘ Any language ∘ Ongoing study (e.g., living systematic review, early findings)

## 4 SEARCH METHODS AND SOURCES

The database search strategy will combine terms for the ‘oldest old’, such as centenarians, nonagenarians, octogenarians, ‘oldest old’, ‘fourth age’ and ‘very old’ with terms, or validated filters, for systematic reviews and terms, or validated filters such as those for randomised controlled trials and qualitative studies. A combination of controlled vocabulary and free text terms will be used. We will search MEDLINE (via Ovid), Embase (via Ovid), APA Psycinfo (via Ovid), HMIC (via Ovid), Social Policy and Practice (via Ovid), Ageline (via EBSCOhost), ASSIA (via ProQUEST), CINAHL Complete (via EBSCOhost), Epistemonikos (via www.epistemonikos.org), the Cochrane Database of Systematic Reviews (via the Cochrane Library, Wiley), Campbell Systematic Reviews (via Wiley) and the CENTRAL database (via the Cochrane Library, Wiley).

To capture grey literature we will search: A&HCI (Arts and Humanities Citation Index), ESCI (Emerging Sources Citation Index), CPCI‐SSH (Conference Proceedings—Social Science and Humanities), CPCI‐S (Conference Proceedings Citation Index‐Science), SCI‐EXPANDED (Science Citation Index Expanded), SSCI (Social Sciences Citation Index)—all via Web of Science, and ProQuest Dissertations and Theses Global. We will search the websites of key organisations such as (Centre for Ageing Better, WHO, The King's Fund, Independent Age) for studies contained within reports.

We will identify studies not caught by the database studies searches by carrying out forwards and backwards citation chasing of included primary studies, examining the included studies within related systematic reviews and hand‐searching any key journals identified during the search process.

We will perform sibling study searches to identify nested qualitative studies of included trials.

For ongoing RCTs we will search in clinicaltrials.gov and the International Clinical Trials Registry Platform (ICTRP), and for ongoing systematic reviews we will search the International prospective register of systematic reviews, PROSPERO. We will not be checking the progress of ongoing studies, other than forward checking whether there has been any studies published from them.

## 5 ANALYSIS AND PRESENTATION

### 5.1 Report structure

The EGM report that will accompany the online interactive map will follow accepted reporting standards for EGM (White et al., 2020): executive summary, background, methods, results, and conclusion. We will present any changes made between the protocol and the final report. The results section will present data on the number of studies included from the database search and provide an overview of the types of study designs by intervention, outcomes, and filters used. We will also provide information on how health equity has been considered in the studies, provide an overview of the main gaps in the evidence, and identify any limitations of this research. The conclusions will provide implications for researchers, policy‐makers, and healthcare providers, and allow us to propose recommendations for future research priorities.

Tables and figures we will include:

Figure: PRISMA flowchart.
Table: Number of studies by study design.
Table: Number of studies by interventions and outcomes.
Table: Number of studies that considered health equity.
Other tables and figures will be included based on coded information for selected filters.
Appendix: Full search strategy used for each database.

### 5.2 Filters for presentation

We will collect details on characteristics that may be of interest to decision makers, as filters for the evidence—such as but not limited to, setting (e.g., hospital, long‐term care, independent living), the country of the study, health condition (e.g., dementia, diabetes, obesity), focus on common geriatric syndrome (e.g., falls, incontinence, frailty, pressure ulcers, delirium), study design (e.g., qualitative study, RCT or systematic review), and quality of the evidence.

We will also be using the PROGRESS‐Plus criteria as filters for the map (https://methods.cochrane.org/equity/projects/evidence-equity/progress-plus). We will identify whether the research question considered any of the PROGRESS Plus criteria and/or whether the included studies reveal anything about inequality, for example, if despite the RQ being for all older people aged 80 years or more (already a protected characteristic) the study/review only ended up including evidence on women or men.

### 5.3 Dependency

When there are multiple reports for a single study, we will treat them as one study. Randomised controlled studies will be captured on the map even if they are included within a systematic review. We accept there may be more than one SR which includes the same RCT. We acknowledge that this will lead to duplication, but the purpose of the map is to demonstrate the breadth of evidence.

## 6 DATA COLLECTION AND ANALYSIS

### 6.1 Screening and study selection

Once the search results have been obtained, six reviewers (BA, AB, JTC, RWE, NO, MR) will independently apply the inclusion and exclusion criteria to a representative sample of citations (e.g., *n* = 50). Decisions will be discussed in a group meeting to ensure consistent application of criteria. This will allow us to clarify the inclusion and exclusion criteria, and revise them where necessary, enabling consistent reviewer interpretation and judgement of the criteria.

After the pilot screening exercise has been completed, two reviewers (BA, AB, JTC, RWE, NO, MR, RG, VG, IL, AM) will independently apply the revised inclusion and exclusion criteria to the title and abstract of each identified citation. We will obtain the full text of papers where either reviewer judges it to meet the criteria, and for those where it is not possible to make a decision using the information in the title and abstract alone. Two reviewers will assess the full text of each record independently for inclusion, with disagreements settled through discussion with a third reviewer. This will include deciding whether each study is a systematic review according to the five criteria detailed above. The study selection process will be detailed using a PRISMA‐style flowchart, with a reason reported for exclusion of each record retrieved at full text (Moher et al., 2015).

The principle focus of any eligible intervention is adults over 80 years of age. For reviews. we will judge this during full‐text screening at the level of the systematic review aims. In the case of systematic reviews which may include studies which do not meet our criteria, that is, they have some studies with older adults who are not defined as >80 years, we will include them if over 75% of included studies are relevant. We will not check primary research studies so if this is not evident from the information reported in the paper the study will be excluded. Similarly, we will not check for duplication of primary studies between reviews as the map is intended to capture the breadth of evidence available.

### 6.2 Data extraction and management

Two reviewers will independently extract data on published and ongoing systematic reviews, randomised trials and qualitative studies related to the population, intervention, comparison, and outcomes. Coding categories for data extraction will be based on our intervention/outcomes framework. In addition, we will collect details on characteristics that may be of interest to decision makers as filters for the evidence—gender (male only, female only, mixed), the country of the study, health conditions (e.g., dementia, diabetes, obesity), study design (e.g., qualitative study, RCT or systematic review), setting (e.g., hospital, long term care, independent living). For qualitative studies we will collect data on who the participants were.

We will also collect details, if reported, on health equity as defined according to the PROGRESS framework—place of residence, race/ethnicity/language/culture, occupation, gender/sex, religion, education, socioeconomic status, social capital and other characteristics associated with disadvantage and vulnerability such as sexual orientation, age and disability (O'Neill et al., 2013). Furthermore, we will examine whether studies assessed the effects of the intervention by gender or any other characteristic of health inequality such as socioeconomic status. For systematic reviews, we will report equity characteristics as described, and will not go back to included primary studies for more details.

### 6.3 Tools for assessing risk of bias/study quality of included reviews

Quality appraisal of systematic reviews and primary studies (randomised controlled trials and qualitative studies) will be performed by one reviewer and checked by a second, with disagreements settled by a third reviewer.

Systematic reviews will be assessed using AMSTAR (Shea et al., 2017). The quality of each review will be categorised as high, medium, low or critically according to AMSTAR guidance.

Primary RCTs will be assessed using the Cochrane ‐ Risk Of Bias V1 Tool (Higgins & Green, 2011). RCTs with be regarded as high quality if they are rated at a ‘low risk of bias’ on all six principal areas (random sequence generation, allocation concealment, selective reporting, blinding personnel, blinding outcome measures, incomplete outcome data). If one area is rated as a ‘high risk’ of bias, this study will be categorised as medium quality, and if 2 or more areas are rated as ‘high risk of bias’, this will be categorised as low quality (Higgins & Green, 2011).

Methodological robustness of the primary qualitative studies will be assessed using the Wallace criteria (Wallace et al., 2004).

### 6.4 Methods for mapping

We will use Eppi Reviewer Web for creating the EGM. Studies will be entered into an interactive evidence map to visually represent the distribution of evidence across health and social care domains. The map will have multiple layers, such that studies can be identified by type of intervention and outcome/experience. The ‘surface’ or initially visible layer of the map will display the extent of systematic reviews, RCTs and qualitative studies in a matrix of broad intervention type versus broad outcome/experience. We are hoping with the new software that we will be able to show five bubbles in each cell (high‐quality SR, low‐medium quality SR, high‐quality RCT, low‐medium quality RCT, qualitative study)—colours will be used to depict the dichotomous nature of quality).

All cells in the matrix will be clickable, leading the map user to the next layer of the map, focusing on the available evidence for that particular intervention and outcome/experience combination. The map user will see a graphical representation of the evidence, in the form of a ‘bubble’ or ‘doughnut’ with dimensions (e.g., bubble diameter) and colours determined by the number, type and quality of studies available. Filters as described above will be available for looking at select groups of the evidence, for example, for all hospital‐based studies or for women only studies.

The evidence and gap map will not:

- Provide summary outcomes or describe the findings of systematic reviews or primary research
- Provide information on the detailed nature of health and social care interventions beyond a basic description of type of intervention and setting
- Provide a synthesis of primary research.

## CONTRIBUTION OF AUTHORS

Content: All of the authors involved bring some area of content expertise to the EGM. Rebecca Abbott, Jo Thompson Coon, Alison Bethel, Morwenna Rogers, Rebecca Whear, Noreen Orr, Ilianna Lourida and Ruth Garside have considerable experience in conducting systematic reviews of both qualitative and quantitative research on older adults. Jo Thompson Coon is cochair and editor of the Ageing Group of the Campbell Library and codirector of the Cochrane Campbell Global Ageing Partnership. Debbie Cheeseman is a consultant geriatric nurse and has worked on systematic reviews of older adults. Vicki Goodwin is a physiotherapist with an interest in older adults and frailty and experience of evidence synthesis. Aseel Mahmoud is a community pharmacist with an interest in older adults and frailty.

EGM methods: Jo Thompson Coon, Alison Bethel, Morwenna Rogers and Rebecca Whear have experience in creating EGM in a variety of health service related topics.

Information retrieval: Alison Bethel and Morwenna Rogers are information specialists who have considerable experience of working on EGMs and systematic searches to inform evidence syntheses. Morwenna Rogers is a methods editor for the Ageing Group of the Campbell Library and a member of the Campbell Information Retrieval Methods Group.

## DECLARATIONS OF INTEREST

All authors report no conflicts of interest.

## PLANS FOR UPDATING THE EGM

Once completed the evidence gap map will be updated as resources permit.

## SOURCES OF SUPPORT

The evidence gap map is funded by the National Institute for Health Research (NIHR) Applied Research Collaboration South West Peninsula (PenARC). The views expressed are those of the author(s) and not necessarily those of the NIHR or the Department of Health and Social Care.

## Supporting information

Supporting information.

## Footnotes

^1^
Initially qualitative studies relating to the experiences of interventions will simply be mapped according to the intervention type. If time, resource and data allow, the qualitative experience data may be coded within the map to show key themes and areas relating to ‘experience’, or if this is not possible, presented separately.
