# Supplementary material for: PROTOCOL: Health and social care interventions in the 80 years old and over population: An evidence and gap map
Source: Campbell Syst Rev. 2023 May 9;19(2):e1326. doi: 10.1002/cl2.1326 (PMC10168690; doi:10.1002/cl2.1326)
Supplement: Supplementary file 1 — Supporting information. [file CL2-19-e1326-s001.docx]

**APPENDIX 1 - EXAMPLE OF FULL SEARCH STRATEGY**

MEDLINE

Database: Ovid MEDLINE(R) ALL <1946 to October 21, 2022>

Search Strategy:

--------------------------------------------------------------------------------

1 *“aged, 80 and over”/ or centenarians/ or nonagenarians/ or octogenarians/ (1753)

2 (oldest and (people* or person* or adult* or old or elderly)).ti. (1450)

3 (oldest adj2 (people* or person* or adult* or old or elderly)).ab. (2827)

4 “very old*“.tw. (4822)

5 centenarian*.tw. (2271)

6 nonagenarian*.tw. (1637)

7 octogenarian*.tw. (3725)

8 (over 85 or over 85s).tw. (2712)

9 (over 90 or over 90s).tw. (16423)

10 (over 80 or over 80s).tw. (13270)

11 (year* or age*).tw. (6686226)

12 8 or 9 or 10 (32023)

13 ((year* or age*) adj2 (over 85 or over 85s or over 90 or over 90s or over 80 or over 80s)).tw. (3586)

14 1 or 2 or 3 or 4 or 5 or 6 or 7 or 13 (18147)

15 randomized controlled trial.pt. (579325)

16 controlled clinical trial.pt. (95078)

17 randomi#ed.ab. (692659)

18 placebo$.ab. (233439)

19 drug therapy.fs. (2540699)

20 randomly.ab. (393888)

21 trial.ab. (620810)

22 groups.ab. (2424322)

23 exp animals/ not humans.sh. (5057996)

24 15 or 16 or 17 or 18 or 19 or 20 or 21 or 22 (5510892)

**25 24 not 23 (4804559) *1**

26 exp Qualitative Research/ (77541)

27 qualitative*.tw. (343848)

28 interview*.tw. (429482)

29 experienc*.tw. (1337023)

**30 26 or 27 or 28 or 29 (1860128) * 2**

31 25 or 30 (6224453)

32 meta-analysis/ or systematic review/ or systematic reviews as topic/ or meta-analysis as topic/ or “meta analysis (topic)”/ or “systematic review (topic)”/ (313708)

33 ((systematic* or systematized or integrative or mapping or rapid or scoping) adj3 (review* or overview*)).tw. (304675)

34 ((evidence or interpretive or meta or quantitative or qualitative or integrative or collaborative or mixed method*) adj3 (synthes* or review* or overview*)).tw. (205514)

35 (“meta-analys?s” or metaanalys?s or metasynth*).tw. (245480)

36 systematic review.pt. (210384)

37 meta-analysis.pt. (169546)

38 (cost* adj3 review*).tw. (3794)

39 (data adj extraction).ab. (29676)

40 (narrative adj (review* or synthes?s)).tw. (27823)

41 (cochrane or evidence report).jw. (16344)

**42 32 or 33 or 34 or 35 or 36 or 37 or 38 or 39 or 40 or 41 (540180) *2**

43 31 or 42 (6517886)

44 14 and 43 (6979)

***************************

*1 Cochrane HSS filter Glanville J, Kotas E, Featherstone R, Dooley G. Which are the most sensitive search filters to identify randomized controlled trials in MEDLINE? J Med Libr Assoc. 2020 Oct 1;108(4):556-563. doi: 10.5195/jmla.2020.912. PMID: 33013212; PMCID: PMC7524635.

*2 did not use validated search filters
